# Trends of adult primary malignant renal tumors over 6 years

**DOI:** 10.12669/pjms.296.3736

**Published:** 2013

**Authors:** Ahmed Khalil Ibrahim

**Affiliations:** Dr. Ahmed Khalil Ibrahim, PhD, Urology Division, Department of Surgery, Mosul College of medicine, Mosul, Iraq.

**Keywords:** Renal tumors, Hematuria, Renal cell carcinoma, Transitional cell carcinoma, Radical nephrectomy

## Abstract

***Objective:*** To assess the demographic and clinico-pathological trends, as well as the management options of adult patients with primary malignant renal tumors over 6 years.

***Methods:*** From July 2003 – July 2009, 55 adult patients with primary malignant renal tumors were treated in two teaching hospitals (Jordan University Hospital in Amman – Jordan and Al-Jumhory Teaching Hospital in Mosul – Iraq). Their data were reviewed retrospectively.

***Results:*** Of the 55 patients, 44 had renal cell carcinoma (RCC), eight patients had Transitional cell carcinoma (TCC) of the renal pelvis, two had Sarcoma and one Patient had primary renal lymphoma. Among patients with RCC: the mean age was 56 years, male to female ratio was 3:1. The most common presenting symptom was hematuria. Most of the patients were treated with radical nephrectomy, the clear cell subtype was predominant and most of the patients **were** diagnosed at stage T2. Regarding patients with TCC: mean age was 57 years, all were treated with radical nephro-ureterectomy and removal of bladder cuff, however (50%) of them developed TCC of urinary bladder within 12 months of radical nephro-ureterectomy.

***Conclusion:*** In our series of patients with RCC: there is a trend toward younger age at Diagnosis. We still encountered locally advanced and metastatic disease with a lower rate of incidentally Diagnosed tumors.With regard to patients with renal pelvis TCC: there are trends toward younger age affection, localized disease and early development of bladder TCC which indicate the importance of strict surveillance protocol.

## INTRODUCTION

Primary malignant renal tumors include a wide range of tumors, among them renal cell carcinoma (RCC) and transitional cell carcinoma (TCC) of the renal pelvis are the most common. Others include Sarcomas, lymphomas and leukemia.

Renal cell carcinoma (RCC) overall accounts for 2% of all adult malignancies.^1^ Worldwide, based on probably incomplete figures, about 270000 new cases are diagnosed per year, and about 116 000 patients die per year.^2^ It constitutes approximately 85% of all primary malignant renal tumors, Approximately 54,000 new diagnoses of RCC are made each year in the United States, and 13,000 patients die of disease.^3^ Nearly 20,000 new cases are detected in the European Union annually.^4^

RCC is primarily a disease of the elderly with typical presentation in the Sixth and Seventh decades of life. There is a male predominance with a ratio of 3:2.^5^ The incidence rates are 10–20% higher In African Americans in both men and women.^6^ Annual mortality-to-incidence ratio with RCC is significantly higher compared to other urological malignancies making it the most lethal urological malignancy.^7^

As for geographical variation, the rates are low in Asian countries and high in the USA and Canada. The incidence has increased more than 30% over the past two decades.^8^

It is generally postulated that the increased incidence rates reflect earlier diagnosis at an earlier stage, largely due to more liberal use of radiological imaging techniques. However, advanced disease has also been diagnosed more frequently and the mortality rate has increased as well.^6^

## METHODS

From July 2003 to July 2009, Data were collected retrospectively from the records of adult patients with primary malignant renal tumors presented to Jordan University Hospital and Al-Jumhory Teaching Hospital these data include: age, sex, anatomical side, presenting symptoms, diagnosis, metastasis if any, therapeutic options, histopathological subtype and tumor stage.

The numbers of patients who had full data and fulfill these criteria were 55 patients. The study was approved by the authorities of department of surgery in Mosul College of medicine.

## RESULTS

A total of 55 adults with primary malignant renal tumors presented to us **over** 6 years, 41 (74.5%) were males and 14 (25.5%) females, of those patients 44 had RCC (80%), 8 patients had TCC of the renal pelvis (14.5%), 2 had sarcomas (3.6%) and one had renal lymphoma (1.8%) 

Regarding those with RCC: some of the patients characteristics are shown in Table-I, The main presenting symptom was hematuria followed by loin pain and was incidental in 7 only, Constitutional symptoms (Nausea, weight loss, malaise, fatigue, fever and night Sweating) in 6 patients and metastatic symptoms (bone Pain, cough, hemoptysis and shortness of breath) in 2 patients (4.5%) (Tabkle-I). Only 8 /44 (18.2%) patients had palpable abdominal mass.

**Table-I:** The clinical presentation of patients with renal cell carcinoma

Hematuria	39%
Loin pain	27%
Constitutional	14%
Incidental	16%
Metastatic	4%

**Table-II:** Characteristics of patients with renal cell carcinoma

Patients Number	44	
Male:Female	33/11	3:1
Age(Years)	21 -80 Range:	Mean:56
Side	Right54%	Left46%
Localized Disease	30 (68.2%)	
Metastatic	14 (31.8%)	
*Histopathology*		
Clear Cell Carcinoma	35 (79.5%)	
Papillary	5 (11.3%)	
Chromophobe Cells	2 (4.6%)	
Sarcomatoid	1 (2.3%)	
Collecting Duct (Bellini)	1 (2.3%)	
*Fuhrman nuclear grade*		
I and II	29 (65.9%)	
III and IV	15 (34.1%)	
*TStage*		
T1	6 (61.6%)	
T2	16 (44.5%)	
T3	9 (25%)	
T4	5 (13.8%)	

Ultrasound was the initial diagnostic tool in all patients followed by abdominal computed tomography (CT) Scan and chest X-ray. While MRI, chest CT.scan and bone isotopes scan were done upon clinical suspicion. Selective angiography only done in few patients.

Metastasis at presentation was noticed in 14/44 (31.8%) of our patients, the most common metastatic site was the lung in 8 /14 (57.1%), Bone in 5/14(35.7%), liver in 4/14 (28.6%), brain in 2/14 (14.2%), adrenals in 2/14 (14.2%) and skin in 1/14 (7.1%).

Considering the management of those patients: 36/44 (81.8%) underwent Radical Nephrectomy, among them 6/36 (16.7%) had metastasis (cytoreductive nephrectomy), only one patient had renal vein involvement and one patient had simultaneous solitary lung metastasis resection. An adrenal gland excision was concomitantly performed in 41.7% of the cases when there is extensive renal tumor, upper pole tumor or radiological suspension of adrenal metastasis.

While the other 8/44 (18.2%) were treated non-surgically: 4 of them were treated with targeted therapy (sunitinib), 2 with Interferon-α, one patient treated with Thalidomide and one patient was interminal stage and only palliative treatment given.

Regarding histopathology: conventional clear cell carcinoma was the most common type, The most frequent tumor stage according to the TNM classification system was T2 (Table-II). Positive lymph nodes involvement was found in 6/36 patients (16.7%).Renal biopsy guided by CT. Scan done in 7 patients with metastatic RCC, the other patient had skin nodules over his back proved by skin excisional biopsy to be metastatic RCC. Two patients had end stage renal disease (ESRD) with acquired renal cystic disease (ARCD) who developed RCC.

As regards patients with TCC of renal pelvis: 7/8 were males and one female, mean Age was 57 years (49-62); all had renal pelvic TCC, the main presenting symptom was hematuria in all patients , intravenous urography (IVU)and CT. scan done for all patients, all of them had localized disease and treated with radical Nephro- ureterectomy and bladder cuff removal. Tumor stage was T2 in 5 patients andT1 in 3 patients, tumor grade was well differentiated in 3 patients, moderately differentiated in 4 and poorly differentiated in one patient.

Cystoscopy done for all patients at the same time of the radical Nephro-ureterectomy and showed no bladder growth but within one year 4/8 (50%) of them developed bladder TCC, all were treated with transurethral resection of bladder tumor (TURBT) and 2 of them require further treatment with intravesical immunotherapy.

Two male patients had sarcomas (fibrosarcoma and lieomyosarcoma), treated with radical nephrectomy and proved to have localized disease, one Female Patient presented with left renal mass proved to be renal lymphoma following radical nephrectomy and was referred to oncologist.

## DISCUSSION

Renal cancer in general represent 1.6% and 2% of all cancers in Iraq and Jordan respectively ,in both Syria and Lebanon renal cancers represent 1.8% for each, renal cancer is the 2^nd^ most common urological tumor in Iraq preceded by bladder cancer only, while in Jordan it is the 3^rd^. one preceded by prostate and bladder cancers respectively.^9^

RCC usually occur during the 6^th^ or 7^th^ Decade of life,^7^ however, in our patients the mean age was 56 Years and we have 3/44 Patients (6.8%) younger than 40 years old (21, 25 and 28 years), all of them were females without family history of renal tumors. in a study by Taccoen *et al*. showed that 7.5% of their RCC series were less than 40 years old,^10^

In our series, the male: female ratio was 3:1 (25% female and 75% male) with a Lower female incidence than the known ratio of 3:2.^4^Another study from Jordan showed that the male to female ratio was 3.4:1 and the mean patient age was 54 years.^11^ This is close to our mean age which was 56 years.

The main presenting Symptom was hematuria in 38.6% and loin pain in 27.2%. However in other studies they reported hematuria in 40-60% of patients,^12^ flank pain may be seen in up to 40% of patients and is caused by either bleeding within the tumor or invasion of contiguous tissues.^13^

Constitutional symptoms (weight loss, nausea, malaise, fatigue, fever and Night sweating) in 13.6% while 16% only diagnosed incidentally in our patients, however more than 50% of RCCs are now detected incidentally at earlier stages,^14^ on the other hand, 26% of cases werer diagnosed incidentally in previous Jordanian study^11^ and 14% only diagnosed incidentally in a study by Al-Marhoon,^15^ 2 of our patients (4.5%) presented with metastatic Symptoms (bone pain, dyspnea and cough).

In our series 31.8% had metastatic disease, however up to 40% of patients in other series still present with extra renal growth or metastases.^16^ Among those who present with metastatic disease, 75% have lung metastases, 36% have soft tissue metastases, 20% have bone metastases, 18% have liver metastases, and 8% each have skin and CNS metastases.^17^ However, in our series: most common metastatic site was the lung in 8 /14 (57.1%), Bone in 5/14(35.7%), liver in 4/14 (28.6%), brain in 2/14 (14.2%), adrenals in 2/14 (14.2%) and skin in 1/14 (7.1%). Lymph node enlargement reported in 6/36 (16.7%).

Most of our patients (81.8%) underwent Radical Nephrectomy, among them (16.7%) underwent cytoreductive nephrectomy. In another study radical nephrectomy was performed in 91.6% of the patients.^11^ Although nephron sparing surgery nowadays represent the state of the art in renal cancer management, but none of our patients was offered a partial nephrectomy due to lack of adequate experience with such operations and technical limitations.

Clear cell carcinoma usually constitutes 70-80% in other series^4^and it accounted for only 55.5% in a Jordanian study,^11^ while it represent 79.5% in our series.

Majority of renal tumors are discovered incidentally at earlier stage.^14^ However, we still encountered advanced cases which signify either delayed presentation, improper initial assessment causing delayed diagnosis or the limited availability of the modern imaging modalities.

*Patients with TCC of renal pelvis*: Upper urinary tract urothelial tumors involving the renal pelvis or ureter are relatively uncommon, accounting for about 5% to 7% of all renal tumors,^3^ We had a higher incidence of 8/55 (14.5%), usually men generally are about twice as likely to develop upper tract tumors as are women,^7^however in our series we have one female only.

Mean age was 57 years (49-62 years) which is much younger than the reported age range of 75-79 years.^3^ All of them presented with gross hematuria, Had no metastasis and all were treated by Radical nephro-ureterectomy with bladder cuff removal, however, 4/8 (50%) of patients developed TCC of the bladder within 12 months of the radical Surgery, knowing that all patients had normal cystoscopy done at the time of radical surgery. Those patients were 3 males and one female; all had multifocal TCC in the renal pelvis at the time of radical nephro-ureterectomy, the histopathological examination of the resected bladder tumors showed superficial bladder TCC (Ta –T1) with grade 1-2 differentiation.

Patients with upper tract tumors are at risk for development of bladder recurrence, with an estimated incidence that varies in multiple reports from 15% to 50%.^18^^-^^21^ Most of the bladder recurrences occur within 5 years of the development of the upper tract cancer and 82% of them occur during the first two year.^22^

Primary renal sarcomas, in general, are rare tumors and account for up to 3% of all renal malignancies.^23^ In our series Two (3.6%) male patients (59 and 61 years old) presented with renal mass, underwent radical nephrectomy and the histopathology report proved the presence of fibrosarcoma and lieomyosarcoma respectively and the latter offered further treatment with chemotherapy. One (1.8%) female patient had renal lymphoma following radical nephrectomy for Incidental Renal mass.

## CONCLUSION

In this study there was a trend toward younger age at Diagnosis, especially in females, yet, we have less overall female Incidence. Hematuria and loin pain were the commonest presenting symptoms, we still encountered locally advanced and metastatic disease with a lower rate of incidentally diagnosed tumors which signifies the importance of early consultation and proper assessment of patients with suggestive Symptoms. There are trends toward younger age affection, localized disease and early development of bladder TCC within less than one year following radical surgery in 50% of the patients which indicate the importance of strict surveillance protocol.

## Notes

***Note: ***No Conflict of Interest.
